# Extending the Metabolite Diversity of the Endophyte *Dimorphosporicola tragani*

**DOI:** 10.3390/metabo9100197

**Published:** 2019-09-21

**Authors:** Victor González-Menéndez, Gloria Crespo, Clara Toro, Jesús Martín, Nuria de Pedro, Jose R Tormo, Olga Genilloud

**Affiliations:** Fundación MEDINA, Parque Tecnológico Ciencias de la Salud. Avda. del Conocimiento 34, 18016 Granada, Spain; Gloria.Crespo@medinaandalucia.es (G.C.); Clara.toro@medinaandalucia.es (C.T.); jesus.martin@medinaandalucia.es (J.M.); nuria.depedro@gmail.com (N.d.P.); jose.tormo@medinaandalucia.es (J.R.T.)

**Keywords:** endophyte, secondary metabolites, cerulenin, dendrodolides, epigenetic, adsorptive polymeric resins

## Abstract

Fungi are one of the most prolific sources of microbial secondary metabolites. The production of new metabolites can be achieved using multiple fermentation conditions and by adding small-molecule effectors, including epigenetic modifiers. In the framework of our Natural Product screening programme targeting the discovery of new antimicrobial compounds, we applied multiple fermentation conditions and adsorptive polymeric resins on a large collection of fungal endophytes, to increase and stimulate their fungal secondary metabolite production. During this work the endophytic fungus *Dimorphosporicola tragani* CF-090383 showed antimicrobial activity only when grown in presence of adsorptive polymeric resins. In addition, seven epigenetic modifiers were added to fermentations of this endophytic fungus, in an attempt to activate its cryptic pathways as well as to analyse the metabolites produced under these conditions. *D. tragani* was seen to produce three different mycotoxin dendrodolides when the epigenetic modifiers 5-azacytidine and valproic acid were added to the fermentations, and these compounds were further characterized. However, the fungus produced the fatty acid synthesis inhibitor cerulenin, a molecule not previously described to be produced by this fungal species, only when cultivated in presence of the XAD-16 resin. We have found that the addition of XAD-16 resin resulted in four-fold higher titers in the production of cerulenin when compared to the best production conditions described in literature for the original fungal producer strain, *Cephalosporium caerulens KF-140 (=Sarocladium oryzae)*, in a zeolite-based fermentation, used as an ammonium ion-trapping agent. The production of cerulenin by this strain of *D. tragani*, represents an alternative source for the improved production of cerulenin with better yields.

## 1. Introduction

Fungi are one of the most prolific microbial sources of natural products and are known to produce a wide range of bioactive secondary metabolites (SMs), some of them deleterious to mankind like mycotoxins while others have important pharmaceutical applications, like among others antifungal, antibacterial, antitumoural or immunosuppressant agents [1]. These SMs comprise a high chemical diversity and have been proposed to play crucial roles within microbial communities, such as signal molecules in cell-to-cell communication, defense and virulence factors during pathogenic interactions with plants and animals [2].

Fungal endophytes are widespread in nature and inhabit plants without causing symptoms. They may confer the host plant protection against biotic and abiotic stress factors, producing bioactive SMs against phytopathogens [3]. Up to date, a wide range of bioactive SMs have been described from endophytic fungi [4,5,6], including molecules with relevant antitumoural and antifungal activities such as taxol [7], camptothecin [8], enfumafungin [9], moriniafungin [10] or phaeofungin [11]. However, many bioactive SMs are not produced under standard laboratory culture conditions because the axenic culture is in absence of the host or are only produced in such low amounts that they go undetected during natural product screening [12].

The term “OSMAC” (One Strain Many Compounds) was introduced to describe the use of an array of different culture conditions, with variations of media compositions, and other cultivation parameters (i.e., aeration, shape of culture flasks, temperature and media additives) in order to increase the secondary metabolite diversity produced by a microorganism in response to these perturbations [13]. On the basis of this approach, several culture-based techniques have been described for fungal fermentations to trigger their secondary metabolism, such as the variation of growth media compositions and/or culture conditions, the addition of adsorptive polymeric resins ensuring chemical adsorption of bioactive compounds or non-stable products, the use of chemical epigenetic modifiers or the simulation of microbial interactions by coculturing microorganisms [14]. 

In a recent work with 349 fungal endophytes, we studied the effect of the addition of adsorptive polymeric resins based on styrene-divinylbenzene matrix during fungal fermentations to increase the production titers of some SMs and the chemical diversity of the molecules produced by these fungi [15]. In the same way, the addition of several epigenetic modifiers belonging to both classes of DNA methyltransferase (DNMT) and histone deacetylase (HDAC) inhibitors, in a subset of diverse endophytic fungi was shown to promote the induction of secondary metabolite pathways, otherwise silent under standard culture conditions [16].

The application of these approaches using the right combination of strains and/or production conditions has the potential to induce a higher number of fungal bioactive secondary metabolites than classical approaches. A clear example is the use of nutritional arrays and the addition of several epigenetic modifiers on a diverse fungal population that, in combination with a phenotypic high-throughput screening (HTS), has allowed the identification of new active metabolites [17].

In the framework of our natural product screening programme targeted at discovering new antimicrobial compounds, we applied an OSMAC approach that included the use of adsorptive polymeric resins in fermentations of a large set of fungal endophytes isolated from plants collected in arid areas of Andalusia. As a result of this screening, we observed the production of antimicrobial activities from the extracts of *Dimorphosporicola tragani* CF-090383, when only grown in presence of styrene-divinylbenzene resins [15]. For this reason, we decided to perform an in depth study of this fungal endophyte and assess the effect of multiple nutritional conditions and the addition of metabolic effectors on the bioactive secondary metabolite diversity.

## 2. Results

### 2.1. Strain Characterization

**Figure 1:**
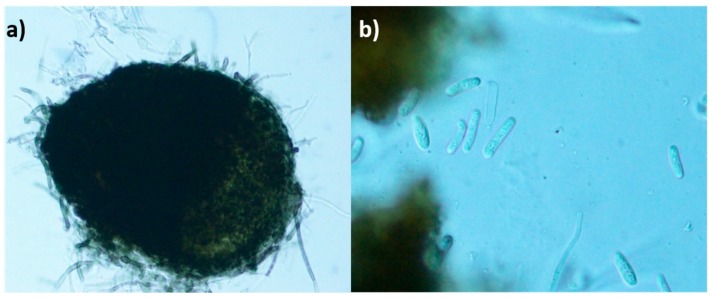
Morphological characteristics of the *D. tragani* CF-090383 strain. (**a**) Cylindrical brown pycnidia. (**b**) Cylindrical hyaline aseptate conidia and paraphyses.

The fungal strain *Dimorphosporicola tragani* CF-090383 was isolated as an endophyte from the disinfected surface of living leaf pieces from the shrub *Arthrocnemum macrostachyum*, collected in the saltmarsh of Cabo de Gata in Almeria, Spain. Due to the pycnidial conidiomata and cylindrical conidia shown in agar culture, the strain was initially identified based on its morphological characteristics as a *Coleophoma*-like fungus (Figure 1). 

The Internal Transcribed Spacer (ITS) sequence of the strain CF-090383 (Genbank accession number MG065838) showed a 99% of similarity with the ex-type strain *Dimorphosporicola tragani* CBS 570.85. In addition, phylogenetic analyses based on ITS and partial 28S confirmed the identification of CF-0903830 strain as *Dimorphosporicola tragani* [15].

### 2.2. Effect of Nutritional Conditions and Adsoprtive Polymeric Resin Addition

**Figure 2:**
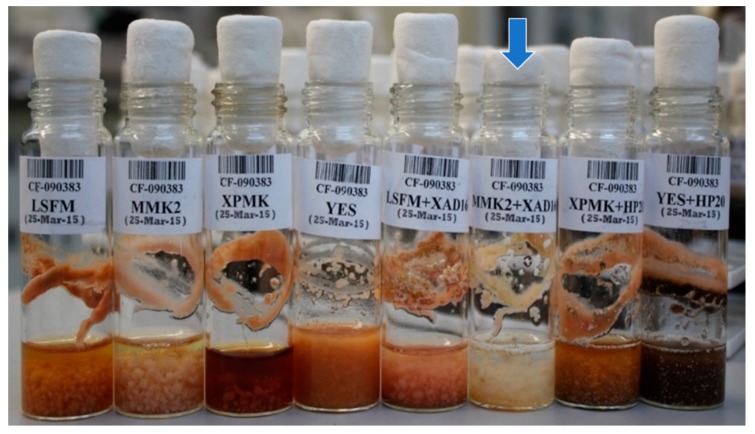
Fungal strain *D. tragani* CF-090383 grown in LSFM, MMK2, XPMK, YES, LSFM+XAD-16, MMK2+XAD-16, XPMK+HP-20 and YES+HP-20 media. After 14 days, phenotypic differences in color, morphology and biomass were detected. The blue arrow shows the fermentation condition (MMK2+XAD-16) that showed the production of antifungal and cytotoxic activity against the panel used in this study.

The strain CF-090383 was grown during 14 days in four different fermentation media in the presence and absence of two styrene-divinylbenzene resins: XAD-16 (Amberlite® from Sigma-Aldrich^TM^, St. Louis, MO, USA), that was added in LSFM and MMK2 media, and Diaion® HP-20 (styrene-divinylbenzene Supelco^TM^, St. Louis, MO, USA), that was added in XPMK and YES media. After 14 days of fermentation, differences in texture, color, morphology and biomass of the cultures were clearly observed among the different conditions (Figure 2).

During the course of the screening, the eight crude extracts of *D. tragani* CF-090383 were evaluated for their bioactivity against two fungal plant pathogens (*Magnaporthe grisea* and *Colletotrichum acutatum*), two fungal human pathogens (*Aspergillus fumigatus* and *Candida albicans*), as well as, the HepG2 liver cancer cell line, showing antifungal and cytotoxic activities only when the fungus was cultivated in the fermentation medium MMK2 supplemented with XAD-16 [15]. In a further attempt to characterize the biological activity profile of this extract in detail, we evaluated the potential to inhibit bacterial growth of the human pathogens *Escherichia coli* MB-2884 and *Acinetobacter baumannii* MB-5973, as well as, the cytotoxic activity on an extended panel of MCF-7 breast cancer and MiaPaca-2 pancreatic cancer cell lines. Results confirmed a strong antimicrobial activity (>90% of cell growth inhibition), as well as, high cytotoxicity (>90% of cell proliferation inhibition) against the three cell lines by the MMK2+XAD-16 fermentation extract.

### 2.3. Chemical Comparison and Dereplication of Dimorphosporicola tragani Fermentation Conditions

**Figure 3:**
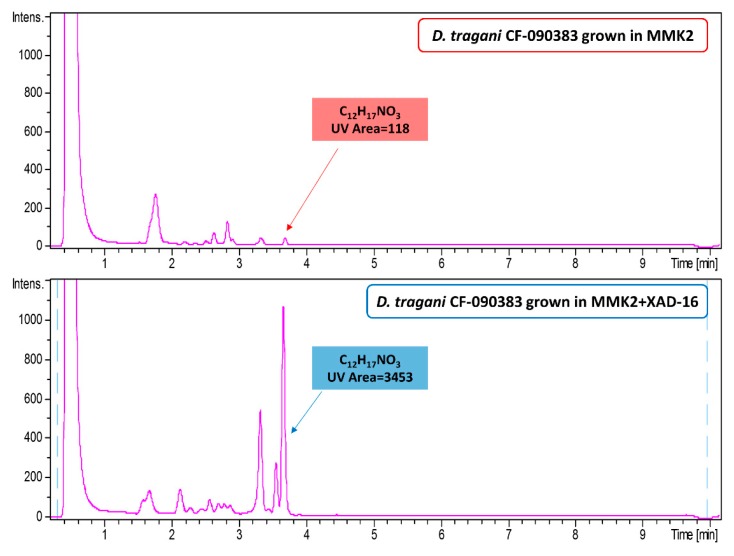
Comparison of HPLC-UV (area at 210 nm) chromatograms of *D. tragani* CF-090383 fermentation extracts from the strain grown in MMK2 medium *vs.* MMK2 medium+XAD-16 resin.

The active extract, corresponding to the fermentation of the strain in MMK2 medium with XAD-16, was analysed by LC-MS against our internal databases of known natural products by fingerprint matching of their HPLC retention time, UV and LRMS (low resolution mass spectroscopy) and was compared with the inactive extract of the fermentation in MMK2 without resin (Figure 3). The comparison of both UV chromatograms at 210 nm showed differential production of some peaks, with larger areas in the fermentation condition with XAD-16 resin. Only one bioactive compound, with the predicted molecular formula C_12_H_17_NO_3,_ could be identified tentatively as cerulenin by HRMS (high resolution mass spectroscopy)_._ The area calculation of this 210nm UV peak was 118 units in MMK2 vs. 3453 units in the same medium containing XAD-16 resin.

### 2.4. Effect of Different Epigenetic Treatments on the Dimorphosporicola tragani Bioactivity Profile

The fungal strain CF-090383 was grown in 40 mL EPA vials containing 10 mL of MMK2 medium and the same medium containing XAD-16 resin, both used as controls, and a 100 mM concentration of seven different epigenetic modifiers (5-azacytidine, hydralazine, quercetin, suberohydroxamic acid (SBHA), sodium butyrate and valproic acid), in order to stimulate its biosynthetic pathways. Ten replicates of each fermentation condition (*n* = 10) were extracted and tested against the three different cancer cell lines previously mentioned (HepG2, MCF-7 and MiaPaca2) in order to evaluate their cytotoxic activity profiles (Table 1). Antitumoural activity could only be observed in the presence of 5-azacytidine and valproic acid, as well as in addition to the XAD-16 resin, previously described.

### 2.5. Untargeted Metabolomic Evaluation of the Differential Bioactive Secondary Metabolite (SM) Profiles of Dimorphosporicola tragani 

Differential untargeted metabolomic studies using Volcano Plot analysis were carried out to compare the potential induction of secondary metabolites in the presence of the epigenetic effectors that could be related to the production of antitumoural activities by the strain *D. tragani* CF-090383. Extracts of ten replicates for each fermentation condition (MMK2 (control), MMK2+XAD-16, MMK2+5-azacytidine and MMK2+valproic acid) were analysed by LC-MS to characterize the pseudomolecular ions of the metabolites produced per production condition [16].

**Figure 4:**
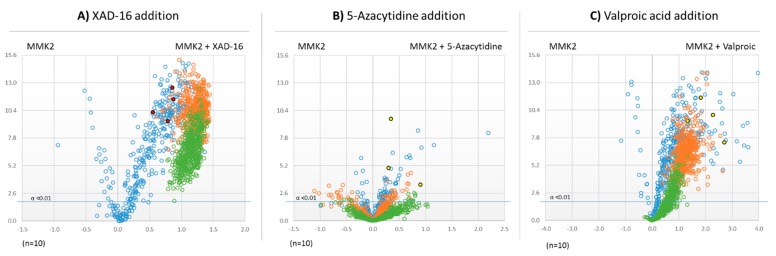
Volcano-plot statistical untargeted metabolomics comparison of the three fermentation additives tested versus their corresponding MMK2 control medium condition: (**A**) when XAD-16 adsorptive polymeric resin was added; (**B**) when the DNMT inhibitor 5-azacytidine was added; (**C**) when the HDAC inhibitor valproic acid was added (*n* = 10; α < 0.01). In blue the pseudomolecular ions *m/z* < 500 Da, in orange pseudomolecular ions *m/z* between 1000 and 1500 Da and in green pseudomolecular ions *m/z* > 1500 Da. Highlighted in other colours are the pseudomolecular ions responsible for the induced antitumoural activity of the compounds (see *Identification of Bioactive compounds* Section). −Log 10 of *t*-test statistical *p*-value in y-axis vs. −log 2 of *m/z* ion masses areas ratios in x-axes.

The Volcano plots resulting from the comparison of the extracts of *D. tragani* grown in MMK2 medium with and without the different inducers highlighted several pseudomolecular ions whose production increased in the different treatments. In the case of the addition of XAD-16 resin, the volcano plot showed a significant increase of the production of pseudomolecular ions with *m/z* above 500 Da when compared to the control (Figure 4A). The addition of 5-azacytidine did not show any significant differences in the number of pseudomolecular ions produced in comparison to the control (Figure 4B). In contrast, the addition of the epigenetic modifier valproic acid significantly induced the production of compounds whose pseudomolecular ions presented *m/z* lower than 500 Da of molecular weight (Figure 4C).

### 2.6. Identification of Induced Bioactive Compounds of Dimorphosporicola tragani

**Figure 5:**
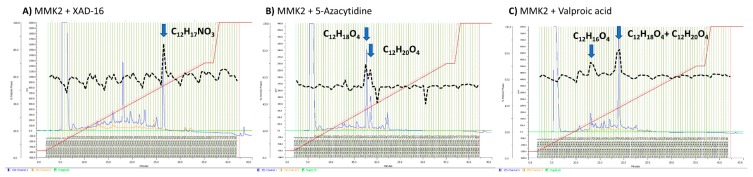
Antitumoural activity profiles (HepG2, MCF-7 and Miacapa-2) marked as a black dashed line overlapping the semi-preparative HPLC fractions for each active fermentation conditions of the strain CF-090383 in MMK2 medium: (**A**) supplemented with XAD-16 resin; (**B**) supplemented with 5-azacytidine, and (**C**) supplemented with valproic acid. Predicted molecular formulas by HR-MS for the main component of each active fraction are indicated in bold.

The extracts of the three active fermentation conditions were analysed by LC-MS and dereplicated against our internal databases of known natural products by fingerprint matching of their HPLC retention time, UV and LRMS (low resolution mass spectroscopy) spectroscopic data. Cerulenin was confirmed in the MMK2+XAD-16 extracts but it could not be detected in the active extracts obtained using the epigenetic modifiers 5-azacytidine and valproic acid. In order to compare and identify the bioactive secondary metabolites produced in these three fermentation conditions and to determine if they were related to cerulenin, we carried out an activity-guided fractionation of their corresponding extracts by semi-preparative HPLC. Upon fractionation we confirmed, one single active fraction from the MMK2+XAD-16 extract whereas two different active fractions were detected in each of the MMK2+5-azacytidine and MMK2+valproic acid extracts. HR-MS (high resolution mass spectroscopy) analysis of the active fractions confirmed the identification of cerulenin in the MMK2+XAD-16 extract and the presence of three additional molecules, with predicted molecular formulas C_12_H_16_O_4_, C_12_H_18_O_4_ and C_12_H_20_O_4_, only observed when the epigenetic modifiers were added to the fermentations (Figure 5).

**Figure 6:**
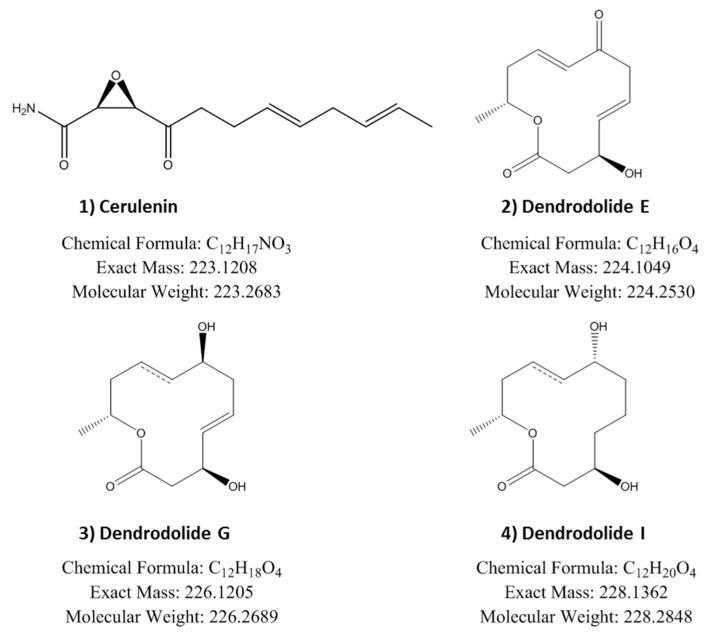
Structure of isolated bioactive compounds (**1–4**).

All active compounds were purified by additional HPLC fractionations. NMR spectroscopy and Mosher analysis allowed the identification of the active compounds as cerulenin and dendrodolide E, G and I (Figure 6 and Supplementary Material).

The antitumoural properties of the four purified compounds were further characterized by testing them as cell proliferation inhibitors against three human cancer cell lines: hepatocarcinoma (HepG2), breast adenocarcinoma (MCF-7) and pancreatic cancer (Miapaca-2). Dose-response curves were performed at concentrations ranging from 0.01 to 100 µM while cells were treated for 72 h to determine the median effective dose (ED_50_) (Table 2). Dendrodolide G was the most potent compound with an ED_50_ of 8.23 µM and 8.25, respectively, for MCF-7 and MiaPaca2, and 12.8 µM in HepG2. Dendrodolide E and I, structurally related to G, showed a similar activity pattern, but with less potency. Cerulenin purified from *D. tragani* showed an effective ED_50_ of 10.14 µM on the MCF-7, whereas the activity was reduced by half when tested against HepG2 and Miapaca2 cell lines.

### 2.7. Understanding the Effect of Adding XAD-16 on the Production of Cerulenin by D. tragani

**Figure 7:**
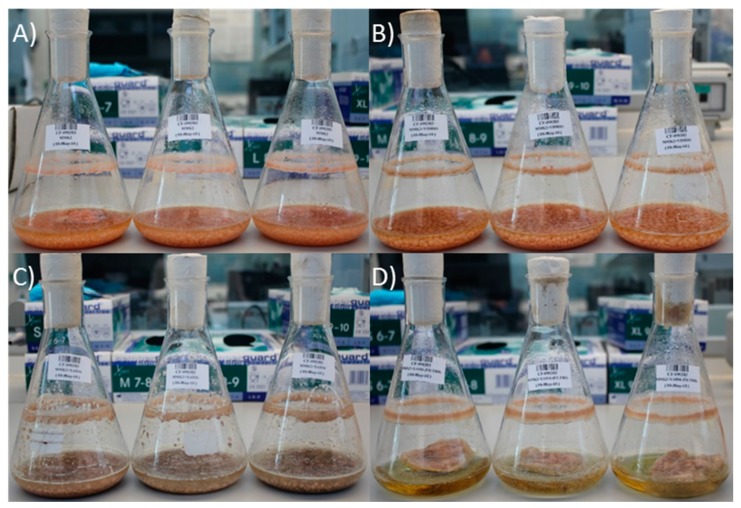
*D. tragani* CF-090383 flask fermentation broths (*n* = 3) after 14 days of incubation: (**A**) MMK2 medium as control, (**B**) with glass beads, (**C**) with free XAD-16 resin and, (**D**) with XAD-16 resin in a cloth bag.

Due to the fact that cerulenin was only produced by *D. tragani* in the presence of the adsorptive polymeric resin XAD-16, we designed an experiment to study cerulenin production conditions. For this purpose, *D. tragani* was grown per triplicate in Erlenmeyer flasks (100 mL) with MMK2 medium (control; Figure 7A), as well as in the same medium containing glass beads as growth support (Figure 7B), XAD-16 resin (Figure 7C), and XAD-16 resin contained in a cloth bag to enable independent resin extraction from the medium (Figure 7D). After 14 days of incubation at 220 rpm and 22 °C all fermentation broths were harvested and extracted. Fermentation broths in the control medium and the medium with glass beads showed the same orange mycelia colour and biomass production (Figure 7A,B). In the case of the fermentation broths with XAD-16 resin, the fungal mycelia were beige for both treatments (Figure 7C,D) but the levels of biomass production were different, as the fungus developed less mycelia on the surface of the cloth bag (Figure 7D) than in the presence of the resin free in the medium (Figure 7C).

**Figure 8:**
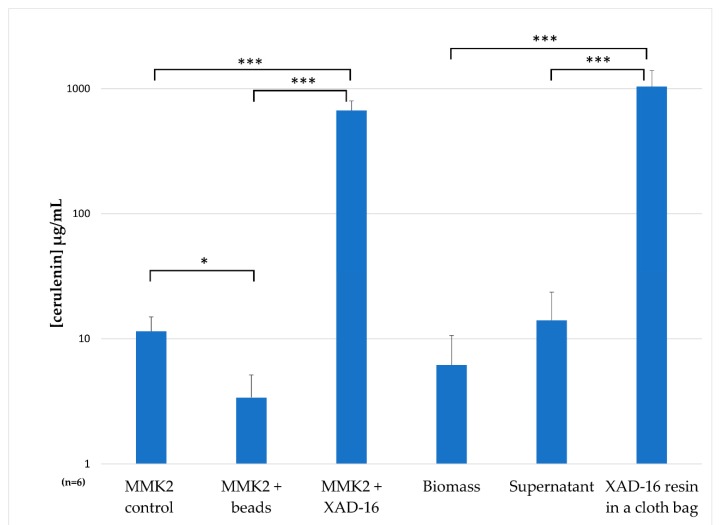
Cerulenin yield production in µg/mL by interpolation of UV area at 210 nm for each fermentation condition tested for the *D. tragani* CF-090383 fungal strain ($\bar{x}$ ± SD; *n* = 6; significant differences calculated by *t*-test).

All fermentation broths were extracted by the addition of an equal volume of acetone. In the case of the resin contained in a cloth bag, the supernatant, the fungal biomass and the resin itself were extracted individually with the same volume of acetone to determine accumulated cerulenin. Cerulenin production yields were determined by interpolation of 210 UV areas for each fermentation condition in a calibration curve of ten-point dilutions using a commercial cerulenin standard from Sigma-Aldrich^TM^. We confirmed a three-fold higher cerulenin production in the presence of XAD-16 (670 ± 128 µg/mL) by *D. tragani* compared to the previously reported zeolite based production condition for *Cephalosporium caerulens* KF-140 strain (250 µg/mL), whereas the two control medium variants tested (control medium and bead addition) were comparable to the original titers of 40.5 µg/mL obtained from the original strain without zeolite, an ammonium ion-trapping agent [18]. Individual quantifications of cerulenin production in the biomass, the supernatant and the resin in a cloth bag showed that the resin XAD-16 captures cerulenin and permits its accumulation during the fermentation process, with only residual amounts of the compound detected in the supernatant and the fungal mycelium. The conditions used resulted in the highest cerulenin yield production obtained so far, in the range of 1035 ± 365 µg/mL, two orders of magnitude higher than the control condition (Figure 8), and four-fold higher than any production condition described in literature for other fungal strains [18].

## 3. Discussion

*Dimorphosporicola tragani* was recently described by Crous as a new species exhibiting *Coleophoma*-like features such as pycnidial conidiomata, cylindrical conidia, and the presence of paraphyses, but that can be distinguished by having conidiogenous cells that can also proliferate percurrently, and dimorphic conidia [19]. The ex-type of this species, the strain CBS 570.85, was isolated from a leaf of *Traganum nudatum var. microphyllum* collected in Mauritania. The similarity of the culture characteristics and the taxonomic position of our endophytic strain of *D. tragani* CF-090383 with the CBS 570.85 strain [15] confirmed both strains as conspecific. 

The application of the OSMAC approach in combination with the addition of adsorptive polymeric resins during the fermentation of the endophytic strain *D. tragani* CF-090383 resulted in the production of the bioactive compound cerulenin. Cerulenin is a commercially available inhibitor of fatty acid and polyketide synthases, with known antimicrobial properties and has also been described as an effective inducer of apoptosis in several cancer cell lines [20]. This bioactive compound has previously been reported from the rice sheath rot pathogen *Cephalosporium caerulens* KF-140 (synonym of *Sarocladium oryzae* [21]), and also from the endophytic fungus *Phomopsis* sp. E99401 [22]. Given that this metabolite was detected to be produced by both a phytopathogenic and an endophytic strain, cerulenin has been suggested to have a potential role in the destruction of host tissue and/or in fending off competing microbes during colonization, after the death of the host [22]. The presence of the helvolic acid and cerulenin gene clusters has been correlated in *S. oryzae* related species with their virulence as plant pathogens [23]. The occurrence of these biosynthetic genes in genomes of several *Aspergillus* species [24], suggests a broader distribution and that other plant-associated fungi may have the potential to produce these toxins.

In the case of the fungal endophyte *D. tragani* CF-090383, cerulenin was produced only when the strain was grown in MMK2 medium supplemented with XAD-16 resin. Several positive effects on metabolite production have been reported when an adsorptive polymeric resin is added to a fermentation medium. Such effects can include the direct chemical adsorption of bioactive compounds or non-stable products, or the removal of products involved in secondary metabolite pathway feedback repression [25] with the potential induction of new secondary metabolites [26]. In order to determine if the increased production of cerulenin observed in the presence the XAD-16 resin could be the result of the activation of an under-expressed biosynthetic pathway, we decided to evaluate the effect of a diversity of seven epigenetic modifiers with different modes of action on the fermentation of *D. tragani* CF-090383. As a result of this study, we confirmed that the extracts of *D. tragani* fermentation broths obtained in the presence of 5-azacytidine and valproic acid exhibited similar antitumoural profiles to the activity profile in the presence of XAD-16 resin.

Untargeted metabolomic analysis by volcano-plots has been proved to be a useful statistic technique to compare and evaluate the effects of different inducers on fungal fermentations [16]. The first analysis of the three different active fermentation conditions of *D. tragani* versus their control medium MMK2, indicated that all of them promoted significant changes (α < 0.01; Figure 4) in the metabolite profile of the fungus. The 5-azacytidine treatment only induced changes in a few pseudomolecular ions, some of them showed later to be related to the antitumour activity. XAD-16 resin treatment highly promoted the production of compounds with molecular weights > 1000 Da, in agreement with the fact that this resin is typically used for the adsorption of large hydrophobic molecules [26]. In the case of the epigenetic modifier valproic acid, the production of compounds with a molecular weight < 1000 Da was highly induced, in agreement with previous publications describing valproic acid treatments to promote mainly small molecules derived from primary metabolism [27].

Cerulenin could only be detected in the XAD-16 extract by LC-MS analysis. In order to determine the origin of the antitumoural activity observed in the presence of 5-azacytidine and valproic acid and determine if they could be related to cerulenin, an activity-guided fractionation was performed, confirming that cerulenin was the only bioactive compound responsible for the activity observed in the presence of XAD-16. In the case of the activities induced by the epigenetic modifiers, we purified and identified three dendrodolides (E, G and I) responsible for the activity, and no traces of cerulenin or any other cerulenin related active metabolites were detected in any of these conditions. Dendrodolides are 12-membered macrolides that were previously isolated from *Dendrodochium* sp., a fungus associated with the sea cucumber *Holothuria nobilis* [28]. Up to 13 different dendrodolides with moderate cytotoxicity against different cancer cell lines have been described from this fungus, and these activities are in agreement with the ED50 obtained for the dendrodolides isolated from *D. tragani* CF-090383. These results support the concept of the broad distribution of several biosynthetic gene clusters within the fungal kingdom that nevertheless remain often cryptic or poorly expressed in laboratory conditions, therefore limiting the detection of these secondary metabolites.

Once confirmed that the epigenetic modifiers 5-azacytidine and valproic acid had no effect on *D. tragani* CF-090383 cerulenin production, we decided to analyse how the addition of the adsorptive polymeric resin XAD-16 could trigger the production of cerulenin during the fermentation process. The glass beads used in the fermentation had a similar size to the XAD-16 resin but presented no chemical absorption capacity, offering only a surface support for the growth of the fungus. Our results allow us to conclude that the production of cerulenin was not due to an increase in the solid surface available to the fungus in the presence of glass beads and the resin, but to the accumulation of the compound in the resin. In fact after extraction and quantification of the cerulenin production from each condition per triplicate, we confirmed that the highest yields of cerulenin were obtained from the resin when it was contained in a permeable cloth bag. These results confirm the chemical adsorption of cerulenin by XAD-16 resin during the fermentation of *D. tragani* in MMK2 and its accumulation, displacing the compound in solution and ensuring an increase in the final production titers of up to 1 mg/mL. This ability of styrene-divinylbenzene resins to adsorb secondary metabolites of interest has been reported during bacterial and fungal fermentations [25]. A similar effect to that observed for cerulenin has been described for the production of rubradirin by *Streptomyces achromogenes* subsp. *rubradiris* where the addition of XAD-16 resin promoted four-fold production titers greater than their fermentation without resin [29].

The maximum production titers described for the original cerulenin producer strain *Cephalosporium caerulens* KF-140 (=*Sarocladium oryzae*) with zeolite, an ammonium ion-trapping agent, are of 250 µg/mL [18]. We have shown that the fermentation of the endophytic strain *D. tragani* CF-090383 in the presence of the adsorptive polymeric resin XAD-16 enables four-fold higher cerulenin production titers compared to the previously described production conditions. In fact, cerulenin production has only been confirmed with our strain and when the conspecific ex-type strain *D. tragani* CBS 570.83 was cultivated in similar conditions, no cerulenin production could be observed (data no shown).

In conclusion, extending the metabolite diversity of the endophyte *Dimorphosporicola tragani* CF-090383 by the addition of 5-azacytidine and valproic acid epigenetic modifiers, has proved to be a successful strategy for the production of several toxic compounds such as dendrodolides in laboratory conditions. In addition, the use of XAD-16 resin has been shown to be a new and efficient alternative solution for the obtention of cerulenin, with better production yields than those previously reported in literature for this antimicrobial mycotoxin.

## 4. Materials and Methods 

### 4.1. Strain Isolation and Characterization.

Standard indirect isolation techniques were followed to isolate the endophyte CF-090383 [15]. The strain was cultured on YM agar (malt extract 10 g, yeast extract 2 g, agar 20 g, 1000 mL distilled H_2_O), to study its macro- and microscopic characteristics. The strain CF-090383 was preserved as frozen conidia and mycelia in 10% glycerol at −80 °C and is maintained in the culture collection of Fundación MEDINA (www.medinadiscovery.es). DNA extraction, PCR amplification, DNA sequencing, and phylogenetic analyses were performed as previously described [15].

### 4.2. Fungal Fermentations.

The fungal strain was initially grown in four 40 mL EPA vials (those that meet the requirements of the US Environmental Protection Agency) containing 10 mL of culture media with different carbon and nitrogen sources (LSFM, MMK2, XPMK and YES media [26]) with and without two adsorptive polymeric resins: XAD-16 (Amberlite® from Sigma-Aldrich^TM^, St. Louis, MO, USA) was added in LSFM and MMK2 media, whereas Diaion® HP-20 (styrene-divinylbenzene Supelco^TM^, St. Louis, MO, USA) was added in XPMK and YES media, both at 3% (*v/v*) and following a previously reported protocol [26]. The epigenetic modifiers were dissolved in DMSO or distilled water and aseptically added to each EPA vial containing 10 mL of MMK2 medium to a final concentration of 100 µM as previously described [16]. 100 mL fermentations were performed in 500 mL Erlenmeyer flasks containing MMK2 medium (control) and were supplemented, before sterilization process, in each case with: a) 3% (*v/v*) of glass beads, b) 3% (*v/v*) of XAD-16 resin and c) 3 % (*v/v*) of XAD-16 resin enclosed in a cloth bag of 70 mm of diameter. After 14 days of incubation, fermentation broths were extracted with acetone, and samples were finally prepared in a final 20 % dimethyl sulfoxide (DMSO)/water solution at two whole broth equivalent (WBE) concentration according to the procedure previously described [26].

### 4.3. HPLC-UV-LRMS Profile Analysis, Metabolomics and Quantification

Samples (2 µL) were analysed by HPLC-UV-LRMS. LC analysis was performed on an Agilent 1200 (Santa Clara, CA, USA), using a Zorbax SB-C8 column (2.1 × 30 mm, 1.8 µm) with guard column, maintained at 40 °C with a flow rate of 300 µL/min and 210 nm UV detection. Solvent A consisted of 10 % acetronitrile and 90% water with 0.01% trifluoroacetic acid and 1.3 mM ammonium formate, while solvent B was 90% acetronitrile and 10% water with 0.01% trifluoroacetic acid and 1.3 mM ammonium formate. The gradient started at 10% B and went to 100 % B in 6 min, kept at 100% B for 2 min and returned to 10% B for 2 min to initialize the system. Mass spectrometry acquisition was performed on an Agilent MSD 1100 mass low resolution spectrometer to generate the metabolomic raw data. Cerulenin production yields were determined by interpolation in a calibration 210 nm UV ten-point dilution standard curve, ranging from 2 to 0.004 mg/mL of cerulenin standard in the same HPLC setup (R^2^ 0.9968). The statistical analyses by t test and metabolomic charts (volcano-plots) were calculated according to the procedure previously described by our group [16].

### 4.4. HPLC-HRMS Database Matching Dereplication of Known Metabolites

A Bruker maXis HR-QTOF mass spectrometer (Bruker Daltonics GmbH, Bremen, Germany) coupled to the previously described HPLC system was used when characterization and identification of specific metabolites was needed. Ionization of the eluting solvent was obtained using the standard ESI sources adjusted to a drying gas flow of 11 L/min at 200 °C and a nebulizer pressure of 40 psig. The capillary voltage was set to 4000 V. Mass spectra were collected from 150 m/z to 2000 m/z in positive mode. Database matching was performed by comparing retention time and exact mass generated with the Bruker maXis of extracted components from the samples under study, with the retention times and exact masses from known metabolites stored in a database acquired under the same exact LC-HRMS conditions [30].

### 4.5. Assay Guided Purification

The mycelium and fermentation broth (100 mL) were extracted by adding acetone (100 mL) and shaking at 220 rpm for 2 h. After filtration, the acetone extract was concentrated under steam nitrogen to a final volume of 100 mL (100% water). The aqueous residue was loaded onto a SP207ss resin column (10 g, 100 × 10 mm) for an SPE extraction and eluted with an acetone. Once the organic solvent was evaporated under nitrogen steam, the residue was solved in 100% dimethyl sulfoxide (DMSO) and filtered at 0.2mM prior to semipreparative fractionation using a Gilson GX-281 322H2 (Gilson Technologies, USA). The extract was subjected to semi-preparative reversed phase HPLC (Zorbax SB-C18 column, 9.4 × 250 mm, 5 µm, 3.6 mL/min, UV detection at 210 and 280 nm) eluting with acetonitrile/water, in a linear gradient from 5 to 100 % acetonitrile in 45 min to yield collection 1.8 mL per fraction every 0.5 min for generating 80 central fractions that were tested for antitumoural activity after evaporation of the organic solvent and prepared at 20 % DMSO in water. Compound identities were determined by HRMS and nuclear magnetic resonance (NMR) dereplication [31]. The antitumoural assay panel was performed according to the described procedure [32].

## Figures and Tables

**Table 1:** Cytotoxic activity of different fermentation conditions against three different cancer lines (HepG2, Miapaca2 and MCF-7). Cell proliferation inhibition percentage values in negative and cell overgrowth percentage values in positive.

Fermentation Condition (*n* = 10)	HepG2 (%) ($\bar{x}$ ± SD)	MCF-7 (%) ($\bar{x}$ ± SD)	MiaPaca2 (%) ($\bar{x}$ ± SD)
MMK2	−13 ± 15	−16 ± 19	−17 ± 23
**MMK2+ XAD-16**	**−100 ± 0**	**−100 ±2**	**−92 ± 24**
**MMK2+ 5-azacytidine**	−31 ± 11	**−55 ± 22**	**−63 ± 11**
MMK2+ hydralazine	13 ± 2	−6 ± 4	−27 ± 6
MMK2+ quercetin	−5 ± 9	−19 ± 7	−31 ± 11
MMK2+ SBHA	−29 ± 14	−34 ±1 6	7 ± 7
MMK2+ sodium butyrate	2 ± 16	2 ± 11	1 ± 8
**MMK2+ valproic acid**	**−100 ± 0**	**−100 ± 1**	**−100 ± 28**

Highlighted in bold are conditions that showed a cell proliferation inhibition higher than 50%.

**Table 2:** ED_50_ (µg/mL) of the purified compounds (**1–4**) against three different human cancer cell lines (HepG2, Miapaca2 and MCF-7).

Compound Purified from *D. tragani*	HepG2	MiaPaca2	MCF-7
Cerulenin	23.08	18.53	10.14
Dendrodolide E	> 40	> 40	10.27
Dendrodolide G	12.8	8.25	8.23
Dendrodolide I	36.10	15.12	11.57

## Supplementary Materials

The following are available online at https://www.mdpi.com/2218-1989/9/10/197/s1, Figure S1: Chromatogram and Spectrum of Cerulenin by HRMS, Figure S2: 1H-NMR of Cerulenin, Figure S3: Chromatogram and Spectrum of Dendrodolide E by HRMS, Figure S4: 1H-NMR of Dendrodolide E, Figure S5: HSQC of Dendrodolide E, Figure S6: Chromatogram and Spectrum of Dendrodolide G by HRMS, Figure S7: 1H-NMR of Dendrodolide G, Figure S8: HSQC of Dendrodolide G, Figure S9: 13C -NMR of Dendrodolide G, Figure S10: Chromatogram and Spectrum of Dendrodolide I by HRMS, Figure S11: 1H-NMR of Dendrodolide I, Figure S12: HSQC of Dendrodolide I.
