# Supplementary material for: Extending the Metabolite Diversity of the Endophyte Dimorphosporicola tragani
Source: Metabolites. 2019 Sep 21;9(10):197. doi: 10.3390/metabo9100197 (PMC6835440; doi:10.3390/metabo9100197)
Supplement: Supplementary file 1 [file metabolites-09-00197-s001.pdf]

Supplementary Information

Extending the metabolite diversity of the endophyte *Dimorphosporicola tragani*

Victor González-Menéndez*, Gloria Crespo, Clara Toro, Jesus Martín, Nuria de Pedro, Jose R Tormo and Olga Genilloud*

[1] Fundación MEDINA, Parque Tecnológico Ciencias de la Salud. Avda. del Conocimiento 34, 18016 Granada, Spain;

* Correspondence: victor.gonzalez@medinaandalucia.es; (V.G-M.); olga.genilloud@medinaandalucia.es; (O.G.) Tel.: +34-958-993-965 (V.G-M. & O.G.)

Received: date; Accepted: date; Published: date

Table of Contents

**Figure S1.** Chromatogram and Spectrum of Cerulenin by HRMS	**S3**
**Figure S2.** 1H-NMR of Cerulenin	**S4**
**Figure S3**. Chromatogram and Spectrum of Dendrodolide E by HRMS	**S5**
**Figure S4.** ^{1}H-NMR of Dendrodolide E	**S6**
**Figure S5**. HSQC of Dendrodolide E	**S6**
**Figure S6**. Chromatogram and Spectrum of Dendrodolide G by HRMS	**S7**
**Figure S7.** ^{1}H-NMR of Dendrodolide G	**S8**
**Figure S8**. HSQC of Dendrodolide G	**S8**
**Figure S9.** ^{13}C -NMR of Dendrodolide G	**S9**
**Figure S10**. Chromatogram and Spectrum of Dendrodolide I by HRMS	**S10**
**Figure S11.** ^{1}H-NMR of Dendrodolide I	**S11**
**Figure S12**. HSQC of Dendrodolide I	**S11**

Cerulenin identification

Cerulenin molecular formula $C_{12}H_{17}NO_3$ was established by HRMS: $[M+H]^+$ m/z 224.128 (calcd for $C_{12}H_{18}NO_3$, 224.130)

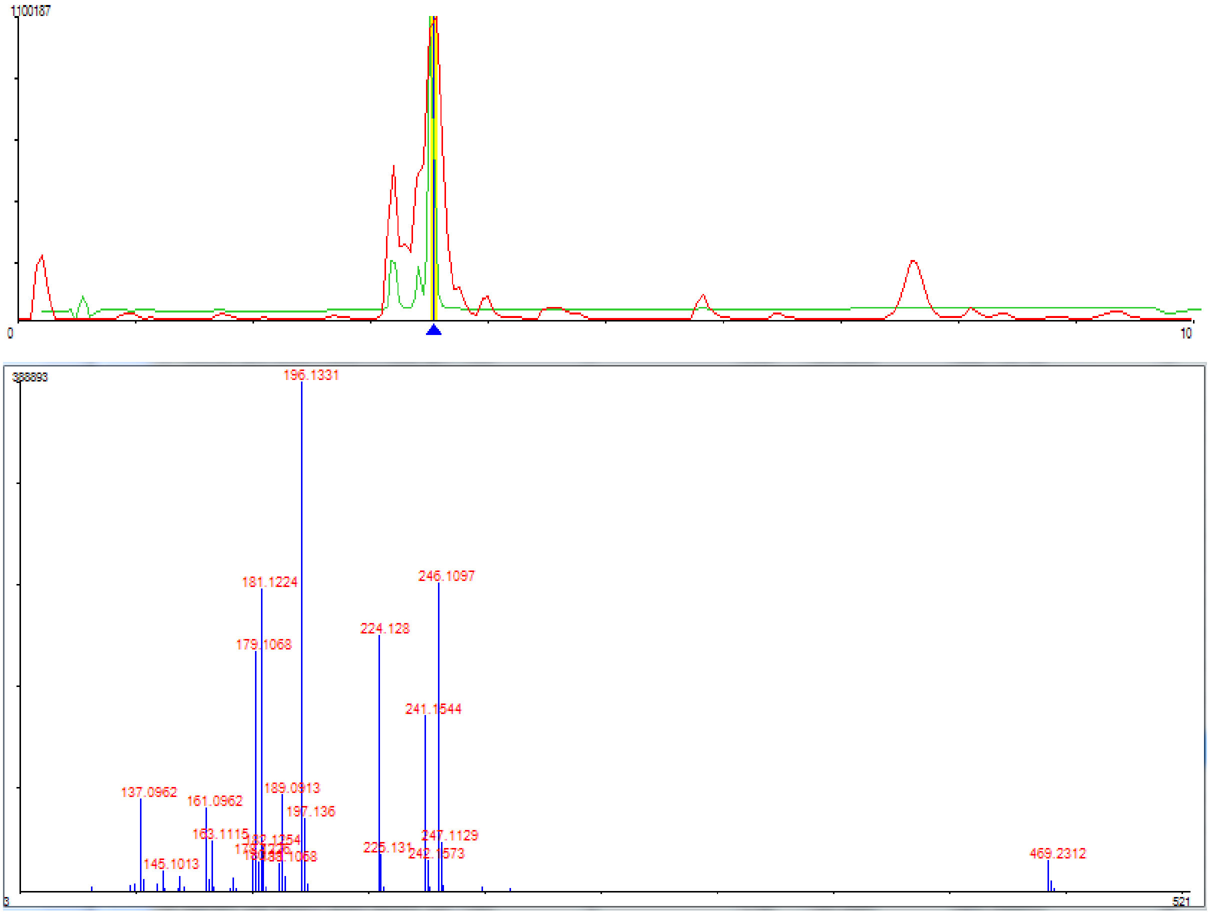

Figure S1. Chromatogram and Spectrum of Cerulenin by HRMS

NMR spectrum of Cerulenin in $CDCl_3$

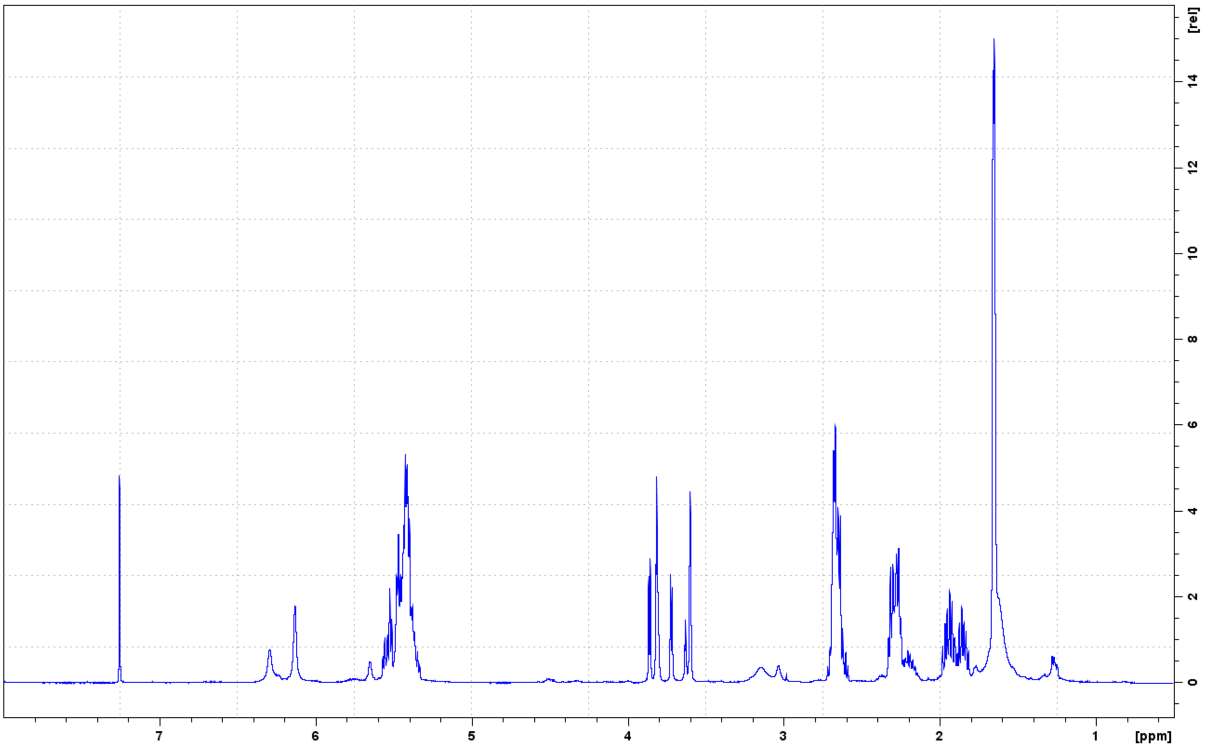

Figure S2. ^{1}H-NMR of Cerulenin

Dendrodolide E identification

Dendrodolide E molecular formula $C_{12}H_{16}O_4$ was established by HRMS: $[M+H]^+$ m/z 225.112 (calcd for $C_{12}H_{17}O_4$, 225.1127)

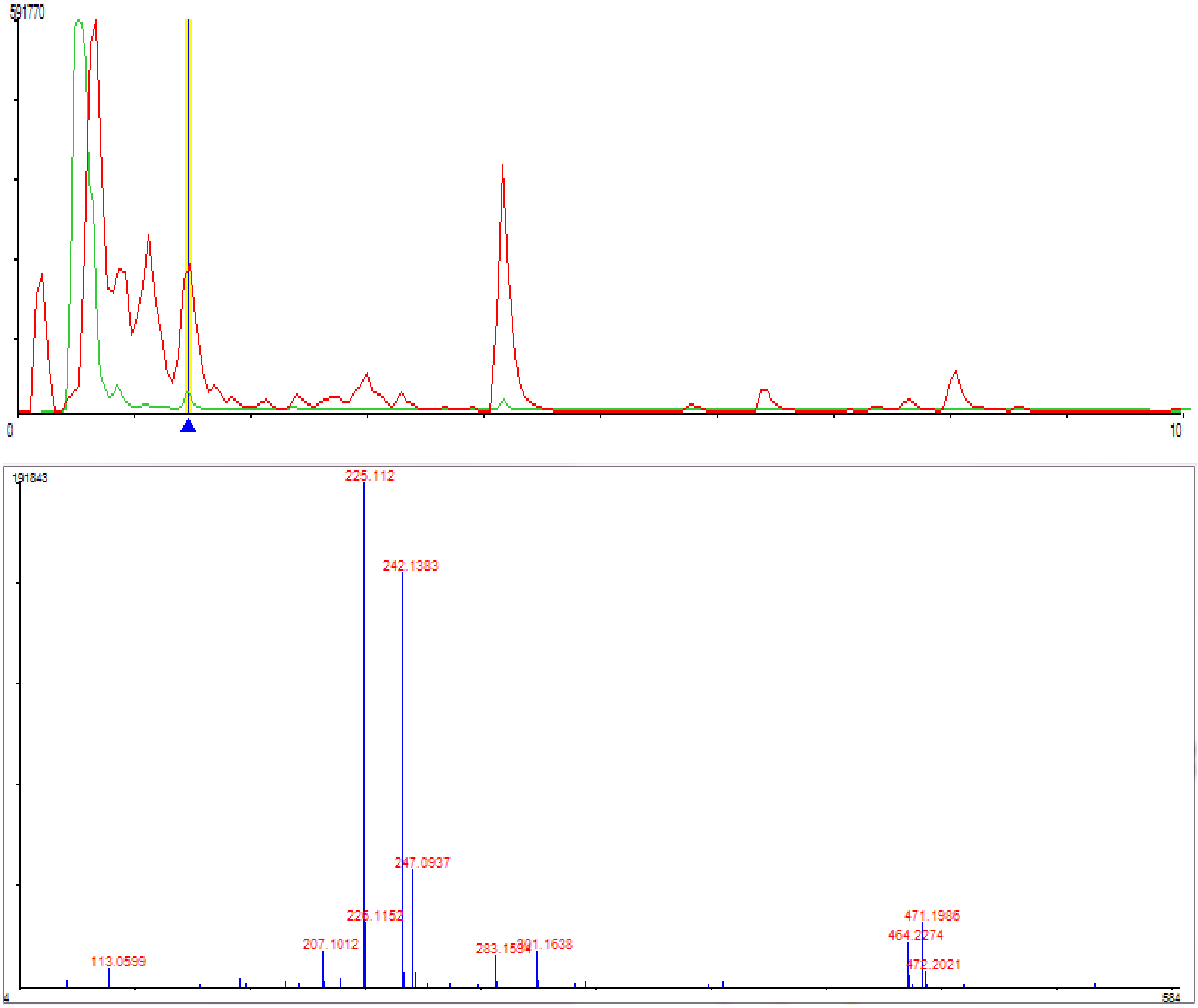

Figure S3. Chromatogram and Spectrum of Dendrodolide E by HRMS

NMR spectra in MeOD

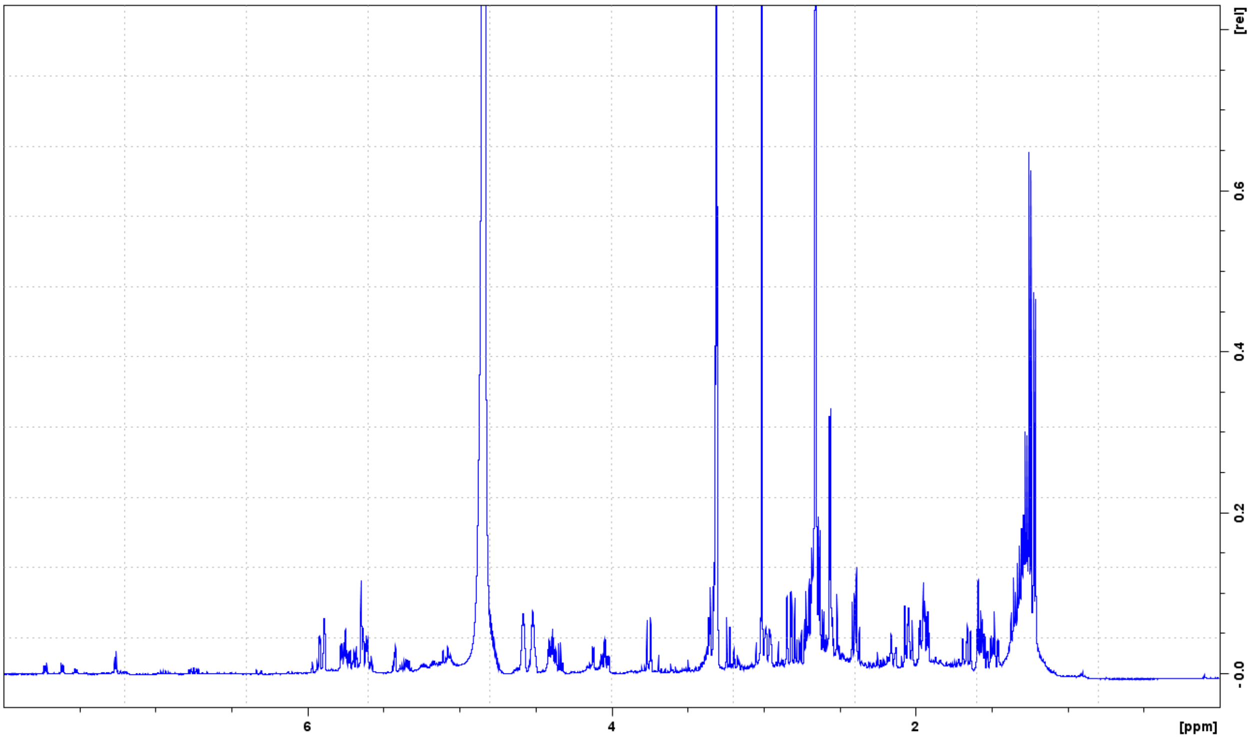

Figure S4. ^{1}H-NMR of Dendrodolide E

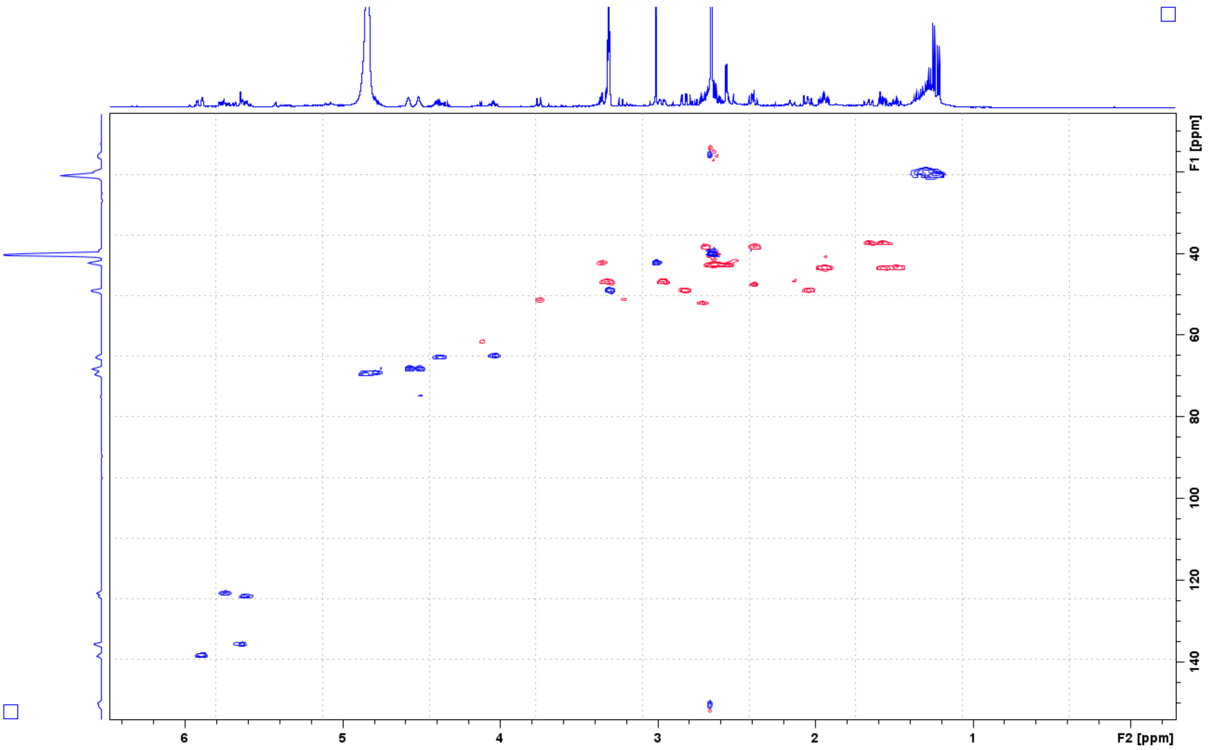

Figure S5. HSQC of Dendrodolide E

Dendrodolide G identification

Dendrodolide G molecular formula $C_{12}H_{18}O_4$ was established by HRMS: $[M+H]^+$ m/z 227.1282 (calcd for $C_{12}H_{19}O_4$, 227.1283)

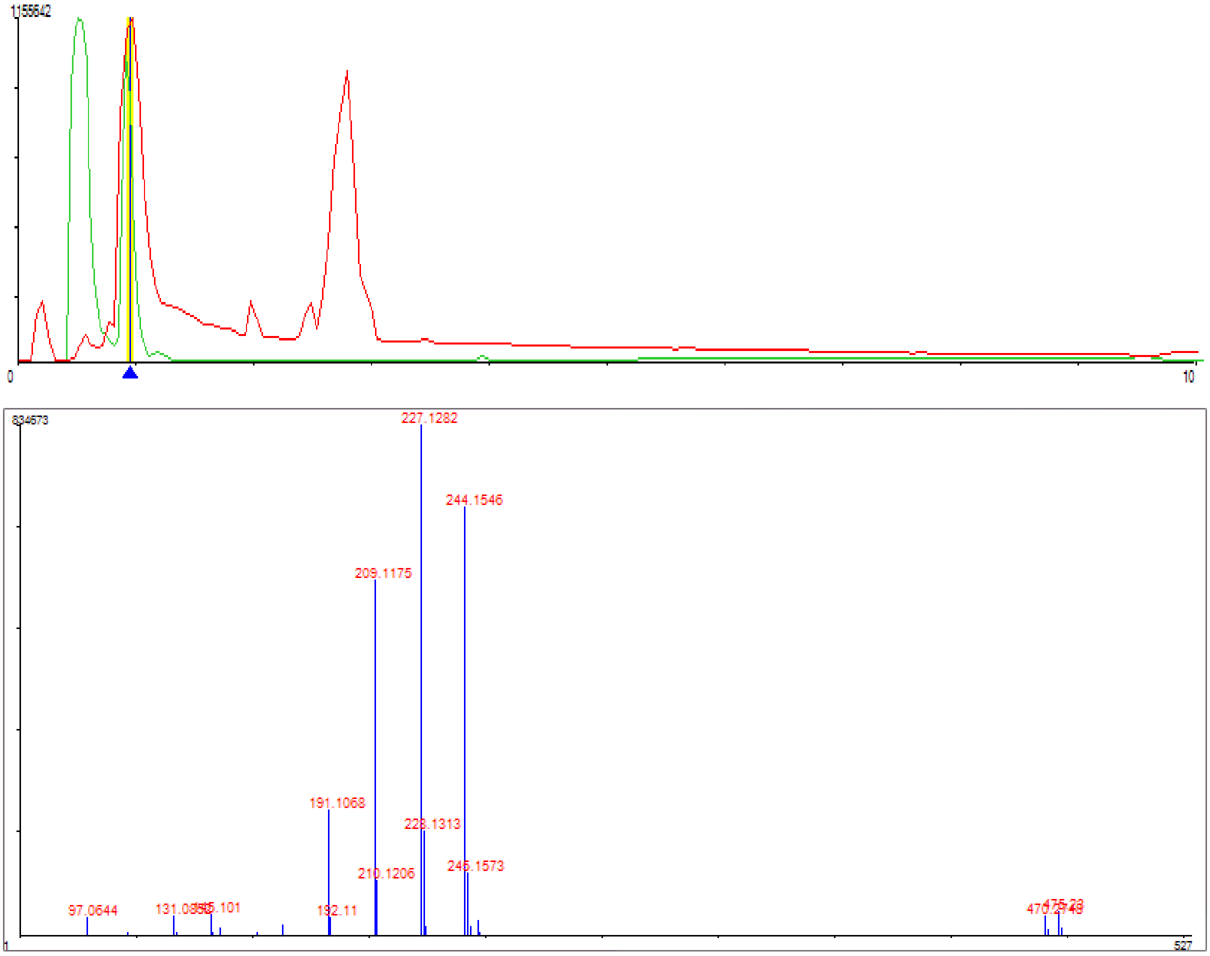

Figure S6. Chromatogram and Spectrum of Dendrodolide G by HRMS

NMR spectra in MeOD

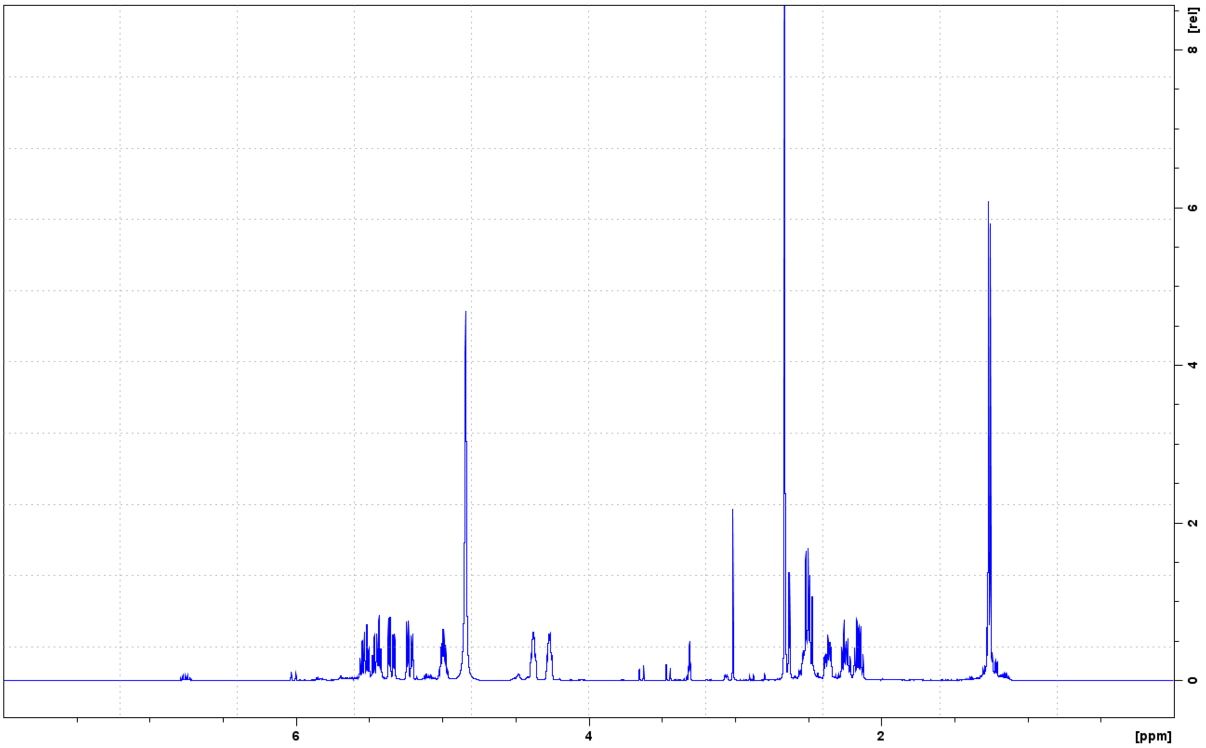

Figure S7. ^{1}H-NMR of Dendrodolide G

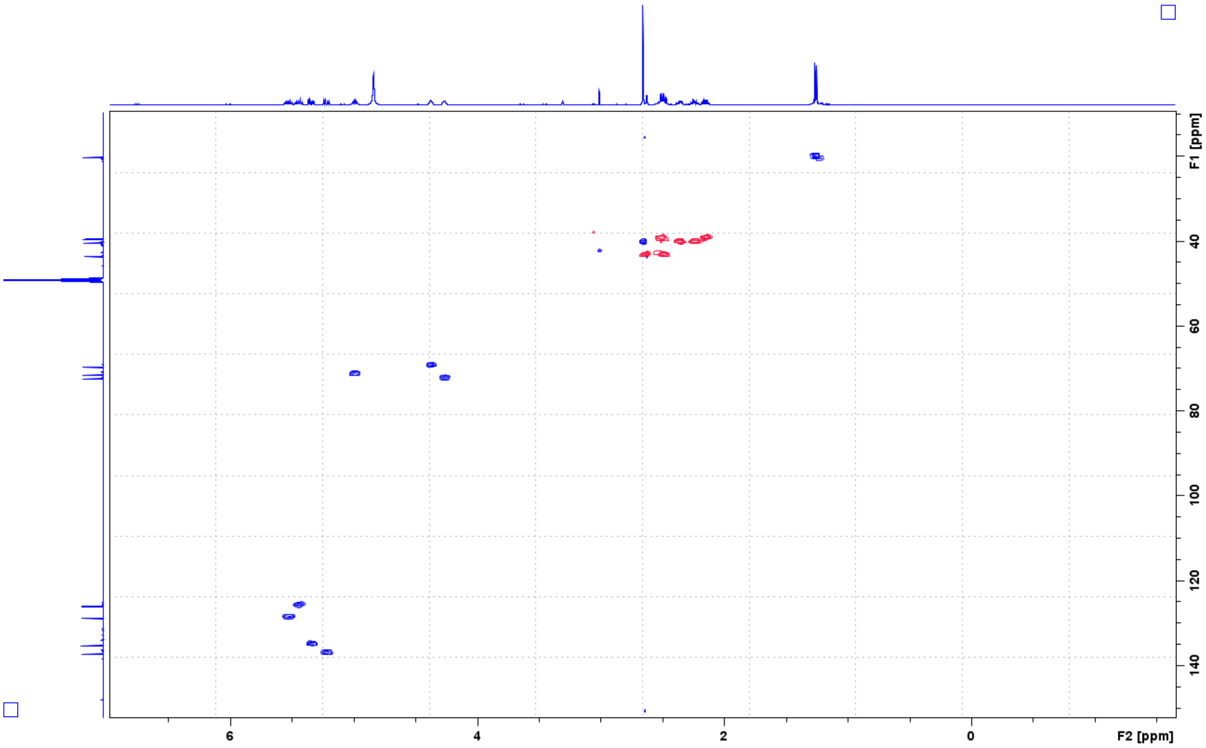

Figure S8. HSQC of Dendrodolide G

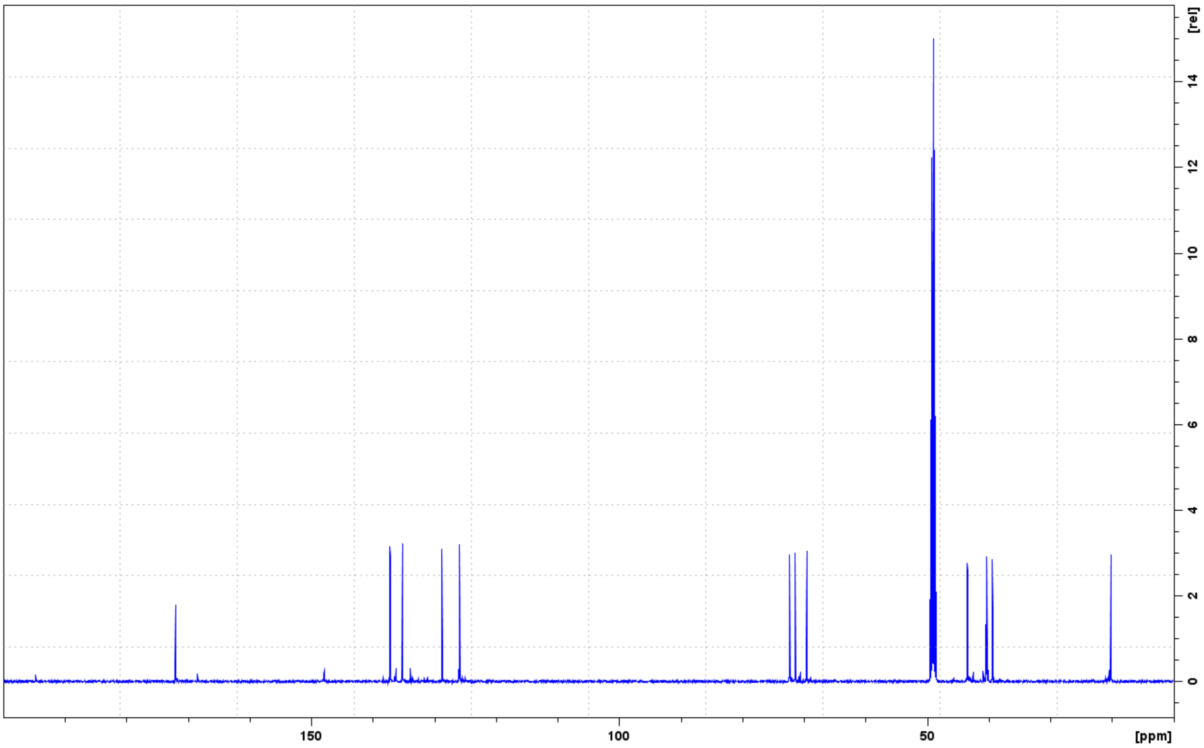

Figure S9. ^{13}C -NMR of Dendrodolide G

Dendrodolide I identification

Dendrodolide I molecular formula $C_{12}H_{20}O_4$ was established by HRMS: $[M+Na]^+$ m/z 251.1247 (calcd for $C_{12}H_{20}O_4Na$, 251.1259)

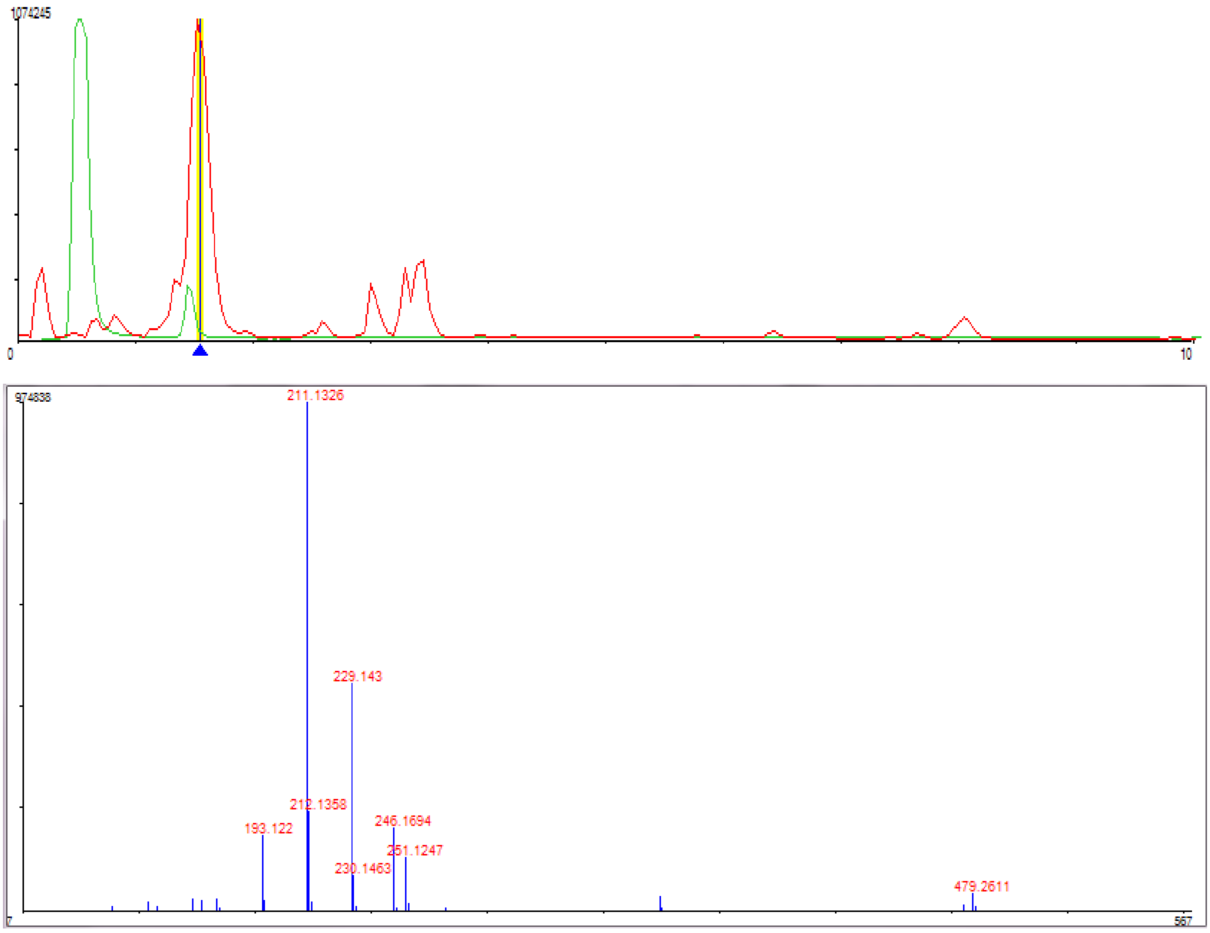

Figure S10. Chromatogram and Spectrum of Dendrodolide I by HRMS

NMR spectra in MeOD

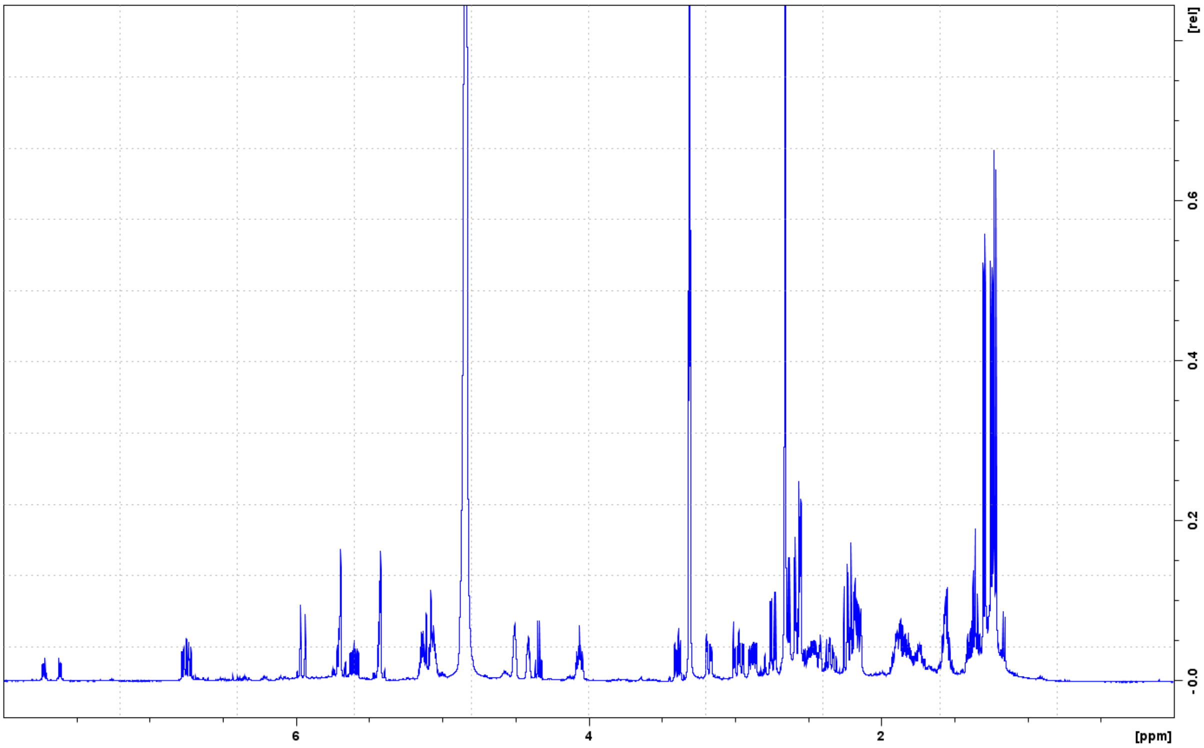

Figure S11. ^{1}H-NMR of Dendrodolide I

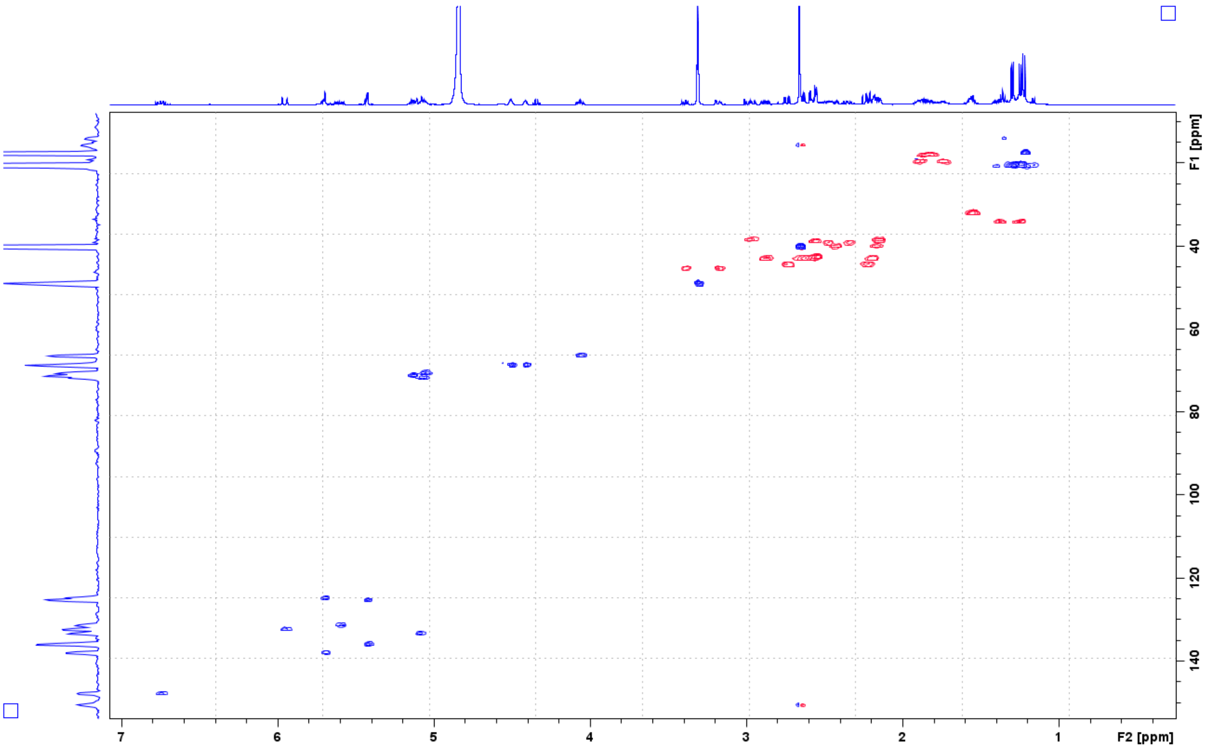

Figure S12. HSQC of Dendrodolide I
